# An Evaluation of Global and Local Tensile Properties of Friction-Stir Welded DP980 Dual-Phase Steel Joints Using a Digital Image Correlation Method

**DOI:** 10.3390/ma8125467

**Published:** 2015-12-04

**Authors:** Hyoungwook Lee, Cheolhee Kim, Jung Han Song

**Affiliations:** 1 Department of Energy System Engineering, Korea National University of Transportation, 50 Deahak-ro, Chungju-si, Chungbuk 27496, Korea; hwlee@ut.ac.kr; 2 Advanced Welding and Joining R&D Group, Research Institute of Advanced Manufacturing Technology, Korea Institute of Industrial Technology, 156 Gaetbeol-ro (Songdo-dong), Yeonsu-Gu, Incheon 21999, Korea; chkim@kitech.re.kr; 3 Metal Forming R&D Group, Research Institute of Advanced Manufacturing Technology, Korea Institute of Industrial Technology, 156 Gaetbeol-ro (Songdo-dong), Yeonsu-Gu, Incheon 21999, Korea

**Keywords:** friction-stir welding (FSW), dual-phase (DP) steel, microstructure, global and local tensile behavior, digital image correlation (DIC)

## Abstract

The effect of the microstructure heterogeneity on the tensile plastic deformation characteristic of friction-stir-welded (FSW) dual-phase (DP) steel was investigated for the potential applications on the lightweight design of vehicles. Friction-stir-welded specimens with a butt joint configuration were prepared, and quasi-static tensile tests were conducted, to evaluate the tensile properties of DP980 dual-phase steels. The friction-stir welding led to the formation of martensite and a significant hardness rise in the stir zone (SZ), but the presence of a soft zone in the heat-affected zone (HAZ) was caused by tempering of the pre-existing martensite. Owing to the appearance of severe soft zone, DP980 FSW joint showed almost 93% joint efficiency with the view-point of ultimate tensile strength and relatively low ductility than the base metal (BM). The local tensile deformation characteristic of the FSW joints was also examined using the digital image correlation (DIC) methodology by mapping the global and local strain distribution, and was subsequently analyzed by mechanics calculation. It is found that the tensile deformation of the FSW joints is highly heterogeneous, leading to a significant decrease in global ductility. The HAZ of the joints is the weakest region where the strain localizes early, and this localization extends until fracture with a strain near 30%, while the strain in the SZ and BM is only 1% and 4%, respectively. Local constitutive properties in different heterogeneous regions through the friction-stir-welded joint was also briefly evaluated by assuming iso-stress conditions. The local stress-strain curves of individual weld zones provide a clear indication of the heterogeneity of the local mechanical properties.

## 1. Introduction

Growing concerns on saving energy and environmental preservations increase the demand for lightweight vehicles. Considerable volumes of advanced high-strength steel (AHSS) sheets have been applied into automotive parts in order to reach the objective of both weight reduction and crashworthiness enhancement [1]. Ferrite-martensite dual-phase (DP) steels are one of the most common AHSS that are currently used in automotive components to meet enhanced government regulation and safety standards. In this combination of two phases, martensite contributes to high strength and a ferrite matrix provides a good elongation that can produce a desirable combination of strength and ductility for applications that require good formability [2]. However, their use has been limited because of narrow welding condition ranges and poor mechanical properties of the weld joint when they are welded with conventional welding processes. To promote the application of AHSS to automobiles, innovations of welding technologies are desired. Friction-stir welding (FSW) is considered as a promising solution.

FSW has been widely studied and increasingly implemented in industrial applications for materials with low melting temperatures such as aluminum and magnesium alloys [3,4,5,6,7,8,9,10]. It shows a more obvious advantage for the high strength aluminum alloys. Currently, many studies have been performed to study FSW mechanisms responsible for the formation of welds [11,12], microstructural refinement [13], effects of parameters on resultant microstructure and final mechanical properties [14,15,16]. However, the research and development of FSW for AHSS has progressed more slowly and remains in early stages. The inherent difficulties of FSW of AHSS are high mechanical loading for plugging and high temperature in the rage of 1000–1200 °C [17,18]. A number of studies have been reported on FSW of steels such as the interstitial free steel [19], carbon steels [20,21,22,23] and stainless steels [24,25]. Recently, with the help of the developments of improved tool materials, FSW for high strength steels have been studied with tensile strength grades between 590 and 1180 MPa. Miles *et al.* [26] investigated the formability of friction-stir and laser-welded DP590 steel sheets. Miles *et al.* [27] also presents FSW results of DP590, TRIP590 and DP980 automotive steels and shows the effect of welding process conditions on weld properties and microstructures. Matsushita *et al.* [28] investigated appropriate welding condition range and the influence of welding conditions on microstructures and mechanical properties of the welds. Friction-stir welding makes it a promising solution for dissimilar material joining. Several studies have been carried out on FSW of aluminum alloy to steel sheets. Uzun *et al.* [29] reported the joint strength of 304 stainless steels, and Al6013 can reach approximately 70% of the base aluminum alloy. Ghosh *et al.* [30] conducted FSW of pure Al to SS304, and the ultimate tensile strength can achieve 82% of Al. Kimapong and Watanabe [31] joined Al5083 with SS400 mild steel and reported maximum shear strength of about 77% of the aluminum base material.Watanabe *et al.* [32] joined SS400 mild steel to Al5083 alloy with a thickness of 2.0 mm. They obtained a maximum tensile strength of 86% of the base aluminum alloy.

While the FSW is utilized for joining similar or dissimilar materials, FSW involves complex heat flow, material movement and plastic deformation. The heat is generated by friction between the tool shoulder and the top of the sheets, and by material movement by the rotation of the pin tool. These thermo-mechanical conditions vary across the joint, introducing a corresponding variation of the microstructure and mechanical properties. Genevois *et al.* [33] determined the tensile properties at different locations of the FSW Al2024 welds using minimized tensile specimens. The tensile properties of the various regions were very heterogeneous. Compared to heat-affected zone (HAZ) and base metal (BM), the welded regions showed a very high hardening rate and a low ductility. From the investigations mentioned above, it should be observed that a qualitative description of the microstructure and strength of the different zones for FSW joint is available [33,34]. However, very little has been done on the plastic deformation characteristic of FSW metals since the local ductility and fracture of FSW joints are more difficult to address than the local microstructure and hardness.

The mechanical performance of any welded component is closely linked to the distribution of properties across the zones affected by the welding operation. Since FSW is a favorable joining process for the production of lightweight components, it is crucial to clarify the local tensile behavior of different weld sub-zones in order to understand the global strength of the welding. Experiment and numerical correlations using finite element techniques are common in studies meant to determine the local and global mechanical responses of friction-stir welding [35,36]. However, there is a lack of investigations in the literature about the systematic analysis of the local deformation behavior and its effect on mechanical properties of the FSW joints of DP steels.

The aim of this paper is to evaluate the global and local tensile properties of friction-stir-welded DP980 dual-phase steels. Specimens with a butt joint configuration were prepared, and quasi-static tensile tests were conducted, to evaluate global and local tensile behavior. Ultimate joint strength and hardening behaviors were examined at a global scale. Local strain distributions in different heterogeneous regions were examined using digital image correlation (DIC) methodology. Finally, local stress strain curves in different regions were successfully analyzed by DIC and mechanical calculations.

## 2. Experimental Procedure

### 2.1. Test Material and Welding Conditions

**Figure 1:**
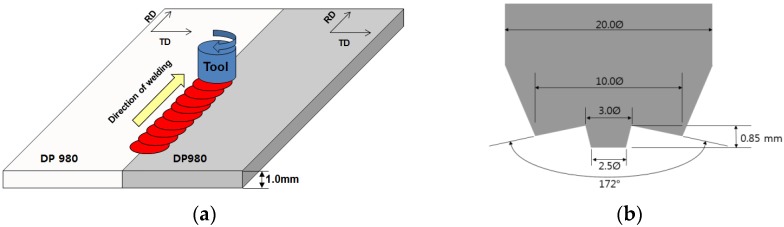
Friction-stir-welding (FSW) of DP980 steel sheet: (**a**) schematic description of FSW process; (**b**) details of cylindrical tool used in FSW process. RD: rolling direction; TD: transverse direction .

The DP980 steel sheet with the thickness of 1.0 mm, as it was received, was used in the present study, and its chemical composition is listed in Table 1. The predicted transformation temperature *Ac*_1_ and *Ac*_3_ of DP980 are 969 and 1133 K, respectively [28]. A schematic illustration of the FSW process is shown in Figure 1a, where a specially designed tool is rotated clock-wise and plugged into the weld line. Figure 1b shows details of the tool with a small diameter threaded probe. Friction-stir-welded butt joints were fabricated with the position-controlled FSW machine (WX-FSW-03, WINZEN, Seoul, Korea). The FSW direction was parallel to the rolling direction of the sheet. The sheets were clamped rigidly onto the top of a steel backing plate and were preheated to achieve a sufficient temperature ahead of the tool to allow the travel. Welding trials were carried out, and optimized process parameters presented in Table 2 were used to fabricate the weld joints free of volumetric defect and lack of severe penetration for further investigation.

**Table 1:** Chemical composition of the DP980 steel used in the present study (wt %).

Elements	C	Mn	P	S	Si	Fe
**Composition (wt %)**	0.065	2.40	0.023	0.004	0.076	Bal.

**Table 2:** FSW parameters used for DP980.

Rotation Speed (rpm)	Axial Force (kN)	Welding Speed (mm/s)	Tilt Angle (Degree)	Tool Geometry		
				Shoulder Diameter (mm)	Probe Diameter (mm)	Probe Height (mm)
600	14	4	2	10	2.5	0.85

### 2.2. Microstructure Analysis and Hardness Measurements

Metallographic samples were polished and went through a chemical etching procedure for the optical microscopy analysis. They were cut off and embedded in an epoxy resin. After a series of grinding and polishing processes, the cross-sections of the weld were etched using an acetic-picral solution. Finally, etched samples were observed and photographed by a scanning electron microscopy (JSM-6500F, JEOL, Tyoko, Japan).

**Figure 2:**
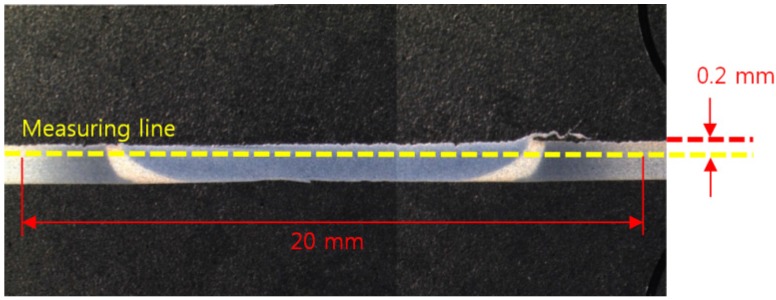
Schemes of hardness measurement positions.

Vickers tests were performed by means of a microhardness tester with a 200 gf load and a dwell time of 15 s. As shown in Figure 2, hardness profiles were measured at a distance of 0.2 mm from the top of the weld with the interval of 0.2 mm. All the microhardness values presented in this study were an average of three series of values taken from the same specimen. The center of the fusion zone was determined carefully after observing the weld geometry under a microscope, and all the indentations were adequately spaced to avoid any potential effect of strain field caused by adjacent indentations.

### 2.3. Tensile Tests

**Figure 3:**
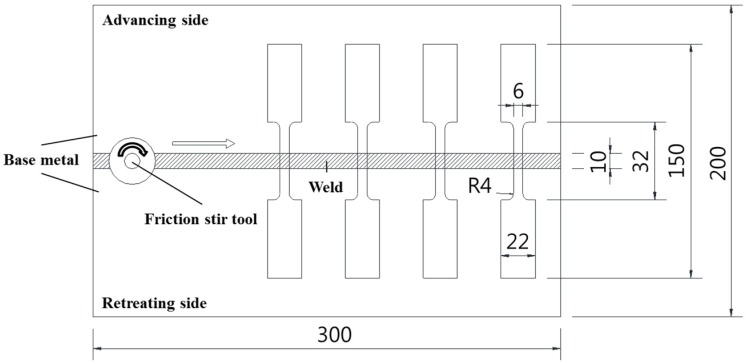
Configuration of FSW sheets and specimen preparation for tensile tests (units: mm).

Uniaxial tensile tests were conducted on the base metal (BM) and friction-stir-welded joints (FSWJ) of DP980 steel. ASTM-E8M sub-sized specimens with the gauge length of 25 mm were used for the tensile tests. The geometry of the tensile test sample is given in Figure 3. Tensile tests were performed at room temperature using a fully computerized universal testing machine (MTS810, MTS Systems Co. Ltd., Eden Prairie, MN, USA). The strain rate tested was 0.001/s. A laser extensometer was used to measure the strain during the test. The ultimate tensile strength and elongation were evaluated. The Kocks-Mecking [37] type of work hardening rates was also analyzed with the tensile results of BM and FSWJ.

### 2.4. Assessment of Local Tensile Properties Using DIC Methodology

In the present study, the local tensile behavior of different welding sub-zones were evaluated by calculating local stress-strain curves from local strain fields registered using the DIC technique. DIC is a non-contact optical technique that can measure full-field two-dimensional or three-dimensional surface deformation. It is a highly responsive method and it requires a simple surface treatment on the test samples [38,39,40]. The DIC algorithm searches for a one-to-one association of points (pixel) in the series of the images taken during testing and calculates the deformation for each stage [41]. A digital image correlation system (ARAMIS 3D, GOM mbH, Braunschweig, Germany) linked to the tensile test machine was used to assess the local strain behavior. During the measurement, trigger signals were sent every second to camera. Management of the entire measurement, evaluation, and documentation procedure was accomplished with the integrated ARAMIS software [42].

## 3. Results and Discussion

### 3.1. Microstructure and Hardness

**Figure 4:**
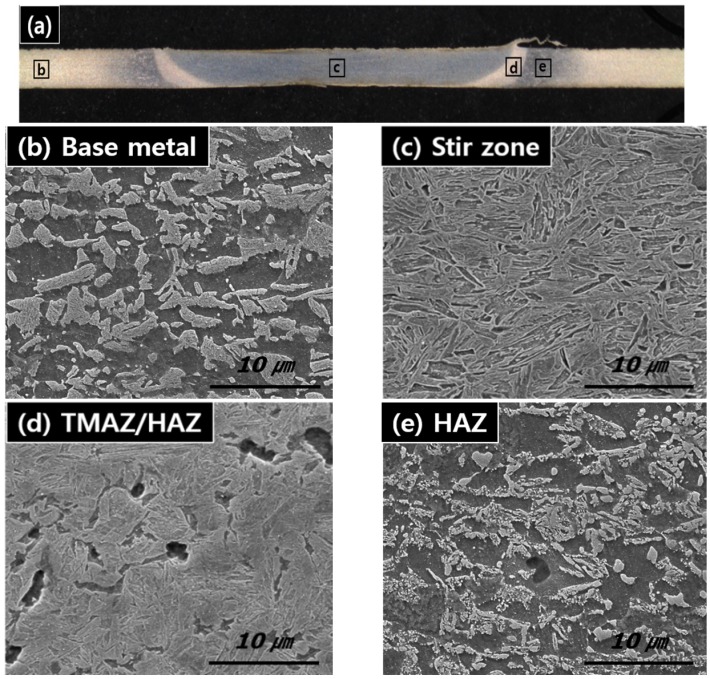
Microstructure of the DP980 FWS joints: (**a**) joint macrograph; (**b**) base metal (BM); (**c**) stir zone (SZ); (**d**) thermo-mechanically affected or heat affect zone (TMAZ/HAZ); (**e**) HAZ.

The overall view of the weld cross-section of the DP980 FSW is shown in Figure 4. It can be seen that the weld cross-section exhibited a heterogeneous structure. It is revealed that the weld joint can be divided into four regions: (1) the stir zone (SZ); (2) the thermo-mechanically affected or heat affect zone above *Ac*_1_ (TMAZ/HAZ > *Ac*_1_); (3) the heat affected zone below *Ac*_1_ (HAZ < *Ac*_1_); and (4) the base metal (BM).

**Figure 5:**
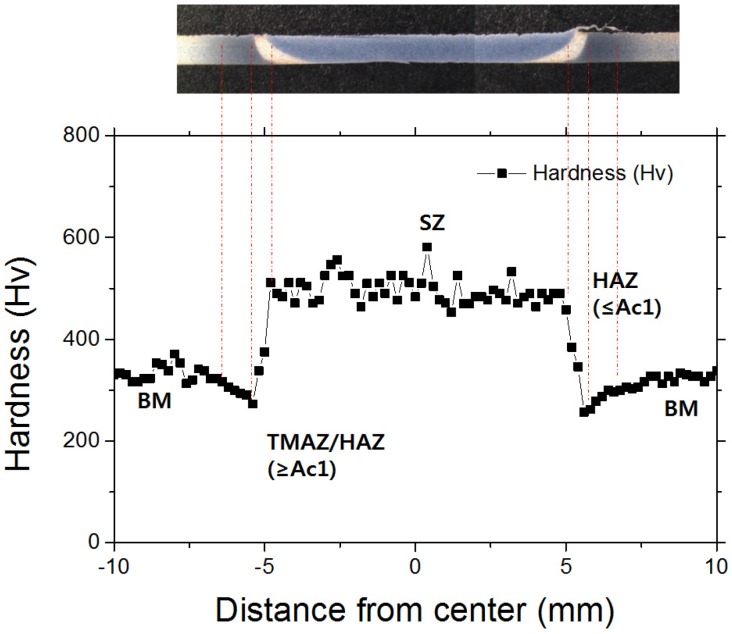
Hardness profile on the DP980 FSW joints.

More detailed microstructure changes are shown in Figure 4b–e. The microhardness profile on the weld cross-section is also presented in Figure 5. The microstructure of the DP980 BM (Figure 4b) was characterized by martensite island in the ferrite matrix. Figure 4c shows the microstructure in the center of the SZ. It seems that SZ was heated up to *Ac*_3_ and rapidly cooled enough for the martensite transformation. This is consistent with the results of previous work on FSW of carbon steel [22] and high strength steel [26,27,28]. Consequently, the microhardness of the SZ becomes higher (average microhardness of 496 HV) than that of the BM (average microhardness of 324 HV). Further from the SZ boundary, in the TMAZ/HAZ, shown in Figure 4d, the peak temperature was between *Ac*_1_ and *Ac*_3_ temperature, which results in austenitization of the carbon-rich martensite, while undissolved ferrite remained unchanged. With increasing distance from the weld centerline, the volume fraction of undissolved ferrite increases, while that of fine martensite decreases. In the HAZ, shown in Figure 4e, the peak temperature seems to be below *Ac*_1_ and the local tempering of the metastable martensite occurs, resulting in the reduction of microhardness. As shown in Figure 5, a microhardness valley was observed in the weld cross-section, in which local microhardness drops below the BM microhardness.

According to the description above, there are local changes in microstructure and microhardness of the DP980 weld joints fabricated by FSW. Hardening in the SZ and softening in the HAZ result in a local strength and ductility change, significantly affecting the overall tensile properties, deformation behavior.

### 3.2. Global Tensile Characteristics

**Figure 6:**
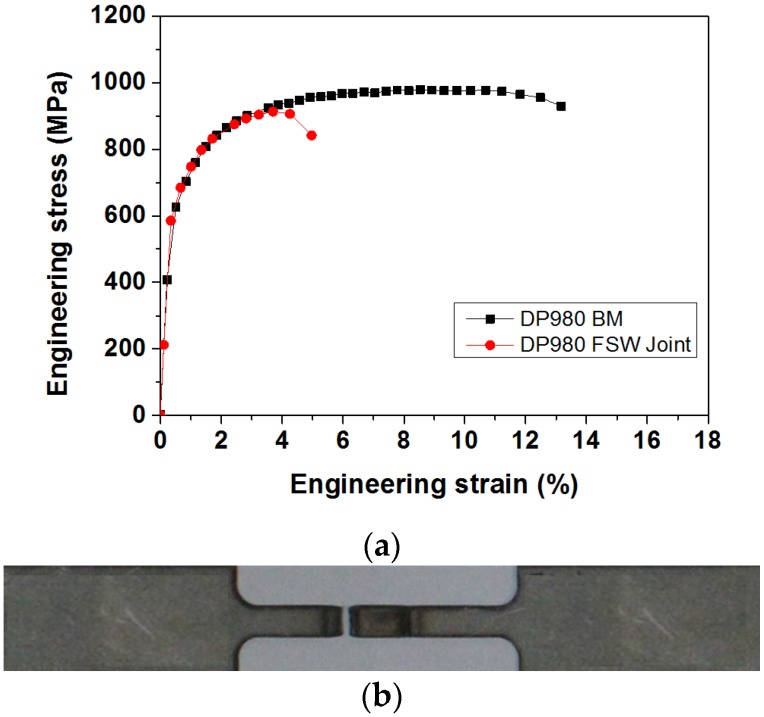
Results for tensile tests of DP980 FSW: (**a**) Engineering stress-strain curve; (**b**) a typical failed sample after tensile test.

Figure 6a shows the engineering stress-strain curves obtained from both the base metal and the FSW joint. All welded samples failed in the HAZ, with an example shown in Figure 6b. The UTS of DP980 FSW joint was observed to be slightly lower than that of the base metal, whereas FSW led to a significant reduction in failure elongation. The ultimate tensile strength of BM is 978.9 MPa, while that of the FSW joint is 912.6 MPa. Thus, FSW joints of DP980 exhibit 93.2% joint efficiency, which results in the hardness drop and softening in the HAZ regions caused by tempering of pre-existing martensite.

**Figure 7:**
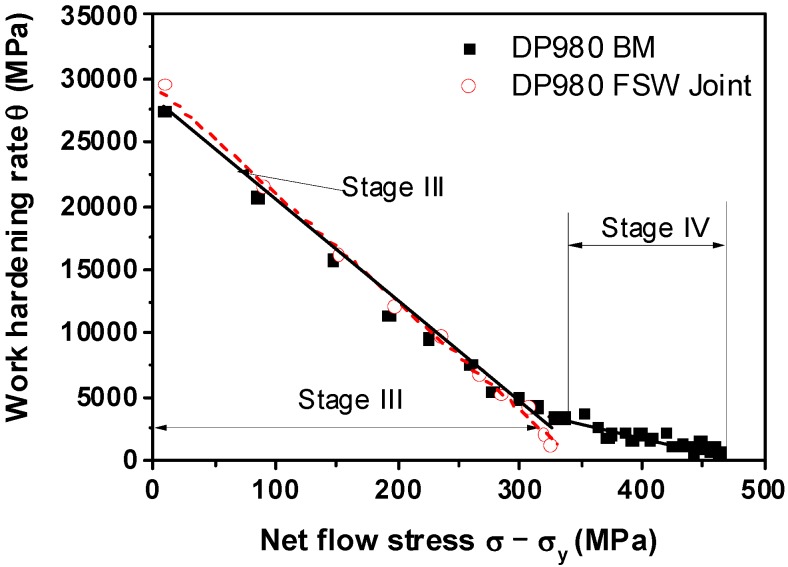
Work hardening rate with respect to net flow stress for DP980 base metal and FSW joint.

Figure 7 shows a Kocks-Mecking [37] type plot of strain hardening rates θ(=dσ/dε) *versus* net flow stress $\left(\text{σ }-{\text{σ}}_{y}\right)$. Initially, the DP980 base metal and the FSW joint showed a similar stage III hardening, *i.e.*, θ decreased almost linearly with increasing net flow stress. When the net flow stress exceeded about 350 MPa, the base metal showed stage IV work hardening behavior despite the small change of θ value, but it was absent in the DP980 FSW joint. The appearance of stage IV hardening in the DP980 base metal was in agreement with the literature [37,43]. The stages of work hardening behavior could be understood as follows: The linear decrease of the work hardening rate in stage III arose from the simultaneous deformation of ferrite and martensite, with an attendant cross-slip of dislocations and dynamic recovery of ferrite [43,44]. In stage IV, the low work hardening rate originated from increased dislocation mobility via profuse cross-slip [43,45].

### 3.3. Local Tensile Characteristics

**Figure 8:**
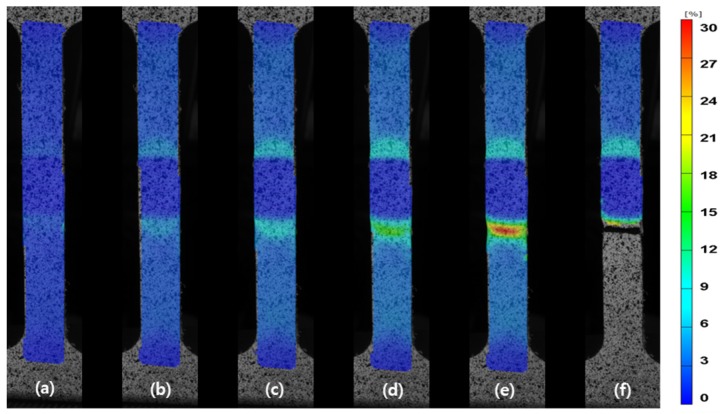
Strain maps resulting from the DIC measurement: (**a**) global strain = 1.0%; (**b**) global strain = 2.0%; (**c**) global strain = 3.0%; (**d**) global strain = 4.0%; (**e**) before failure; (**f**) after failure.

**Figure 9:**
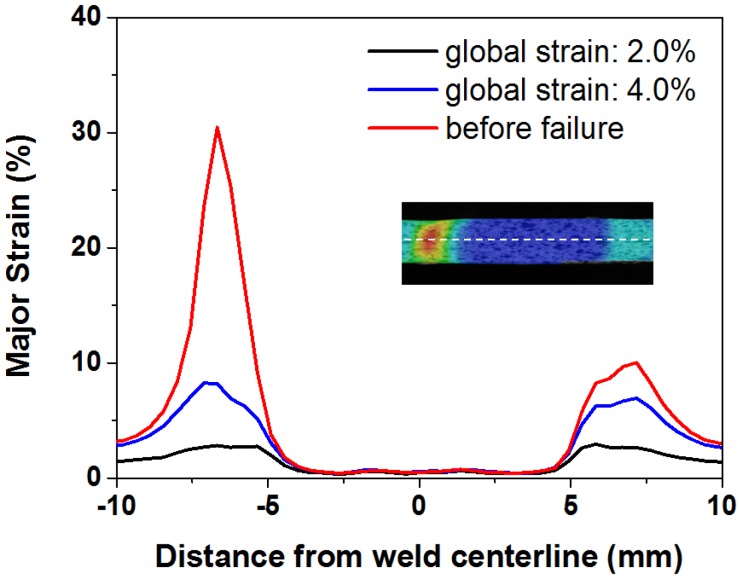
Local major strain profiles along the center of the sample at various global strain.

The weld joint is heterogeneous with different zones that are comprised of various microstructures. Thus, it is expected that the different zones will show different mechanical properties, as expected from the microhardness results. The deformation behavior of the FSW joint during tensile testing was monitored by DIC. Using the ARAMIS system, it is possible to capture the strain maps with digital images at different stages of the tensile testing, as presented in Figure 8. The results clearly show that the strain was heterogeneously distributed across the specimen, mainly due to the microstructural changes in the welded zone. Figure 9 represents the strain distribution along the centerline of the gauge section for various global elongations (2%, 4% and before failure, respectively). Occurrence of plastic deformation and extension of plastic region for the FSW joint are investigated. The plastic strain is uniform across the FSW joint at a global elongation of 2.0%, except the SZ, which shows no obvious changes in the strain. Deformation on both sides of the SZ remains roughly symmetrical until a global elongation of 4%, which indicates that the FSW joint is under a uniform deformation stage. However, this stage is broken, and local necking is observed before failure. The strain localization occurs in the HAZ with a local strain over 29.6%, while that of the opposite side is only 10.5%.

**Figure 10:**
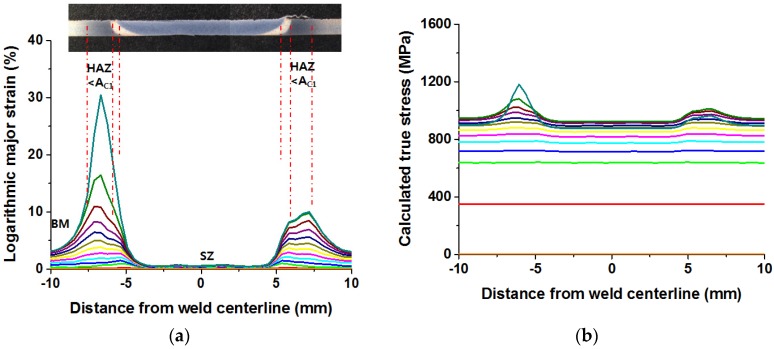
Calculation of localized stress: (**a**) major strain of a section through all load stages; (**b**) stress concentration diagram computed by correlation of the major strain with the load applied in the tensile test.

The strain maps acquired by DIC were processed to access the localized stress-strain responses of the FSW joint. Specifically, the iso-stress condition (e.g., the same global stress at all locations) was assumed for the specimen, a method similar to that employed by other researchers who have studied the local tensile properties in different welded alloys [46,47]. Thus, as shown in Figure 10a, since the local strain values through all load stages during the tensile test is known, the local reduction area in the zone under study during a determined load stage can be estimated with the following equation:

(1) $$A^{i}=A_{0}^{i}\text{exp}\left(-{\text{ε}}^{i}\right)$$

where $A_{0}^{i}$ is the initial cross-section area of the tensile specimen in the evaluation zone, and ${\text{ε}}^{i}$ corresponds to the local axial strain matched by DIC. By dividing the applied load, recorded at the exact time that the DIC system measured the strain, the stress spread along the FSW joint can be calculated using the corrected cross-sectional area ($A^{i}$). The stress diagram computed by this methodology is shown in Figure 10b. Each diagram line corresponds to a single load stage computed through a section that horizontally crosses the region of analysis, as shown in the strain map in Figure 9. If the stress values of each diagram line at a particular point are accumulated, and the corresponding strain values are known, it is possible to create a local stress-strain curve for any specific weld.

**Figure 11:**
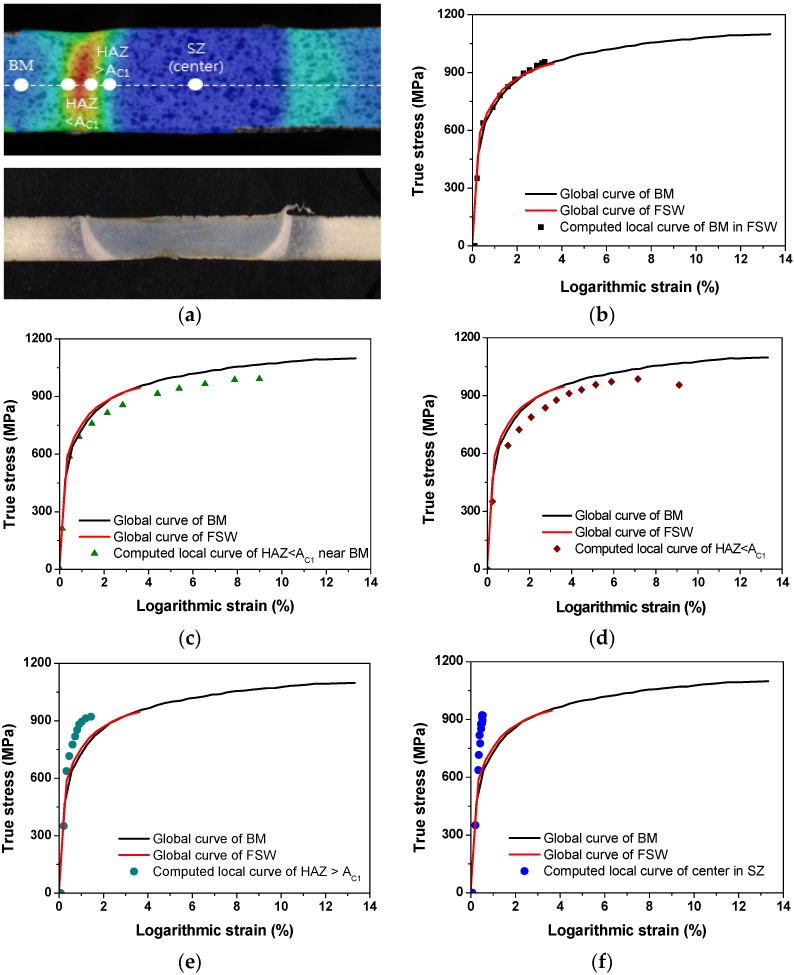
Local stress-strain curves computed by digital image correlation (DIC) for specific microstructural regions: (**a**) measuring points; (**b**) at BM; (**c**) at HAZ < *Ac*_1_ near BM; (**d**) at HAZ < *Ac*_1_ near boundary; (**e**) at TMAX/HAZ < *Ac*_1_; (**f**) at center of SZ.

**Figure 12:**
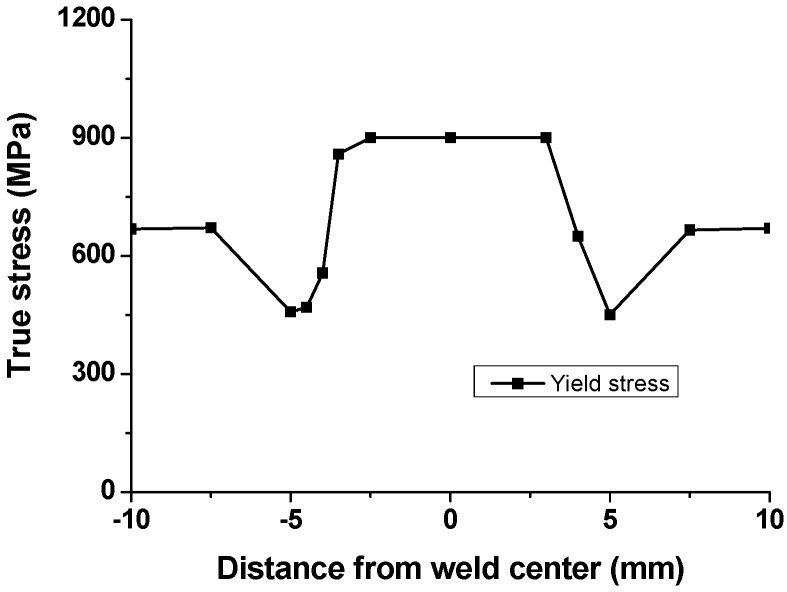
Yield stress obtained by DIC across the FSW joint.

The calculated local stress-strain curves at various regions are presented in Figure 11. In order to verify this procedure using DIC, curves generated for a region approximately 8.5 mm away from the weld center (plotted as solid squares) are compared against the stress-strain curves obtained from the testing for the base materials and FSW joints as shown in Figure 11b. It can be seen that the calculated local stress-strain curve of BM using DIC is in agreement with the curve obtained from the standard global tensile test. The local stress-strain curves of individual weld zones, as shown in Figure 11, provide a clear indication of the heterogeneity of the local mechanical properties. Remarkably, local stress-strain curves at HAZ < *Ac*_1_ shown in Figure 11c–d clearly show softening due to tempering of pre-existing martensite. Figure 12 represents the distribution of local yield stress, which was calculated using local stress-strain curves at various regions. The distribution of local yield stress shows a similar tendency with the hardness profile results. Compared to the BM, the local yield stress increases in the SZ. A local yield valley is observed in the HAZ, in which local yield stress drops below the BM [48].

## 4. Conclusions

In the present study, friction-stir-welded DP980 dual-phase steel was produced. Using FSW samples with a butt joint configuration, the microstructural change after FSW and its effect on the global and local tensile properties were evaluated. The following conclusions can be drawn from the results of the analysis.

1. The friction-stir welding led to the formation of martensite and significant hardness rise in the stir zone (SZ), but the presence of a soft zone in the heat-affected zone (HAZ) was caused by the tempering of the pre-existing martensite.
2. Microhardness reveals that microhardness of the SZ becomes higher (average microhardness of 496 HV) than that of the BM (average microhardness of 324 HV). Furthermore, a microhardness valley is observed at the HAZ in the weld cross-section, in which local microhardness drops below the BM microhardness.
3. The DP980 FSW joint showed almost 93% joint efficiency with the viewpoint of ultimate tensile strength, but showed lower ductility than the base metal due to severe soft zone in HAZ.
4. The base metals of DP980 steel exhibited multi-stage work hardening, whereas their FSW joints showed only a single-stage (or stage III) work hardening where the work hardening rate decreased linearly with increasing net flow stress.
5. Changes in mechanical properties on the local scale were quantitatively assessed by DIC, and stress-strain curves were calculated for different weld regions. Compared to the base metal, the local yield stress increases in the SZ but decreases in the HAZ.
